# What Is the Best Approach to Treating Schizophrenia in Developing Countries?

**DOI:** 10.1371/journal.pmed.0040159

**Published:** 2007-06-19

**Authors:** Vikram Patel, Saeed Farooq, R Thara

## Abstract

Background to the debate: Schizophrenia affects an estimated 25 million people in low- and middle-income countries, with an average lifetime risk of about 1%. The illness is associated with excess mortality from a variety of causes. A 2001 Institute of Medicine report on mental illness in developing countries found that in 1990, over two-thirds of people with schizophrenia in these countries were not receiving any treatment (http://www.nap.edu/catalog/10111.html). The report found no evidence that the proportion of treated people in the developing world had increased since 1990. There is now a debate among mental health professionals in low-income countries over how best to improve patient care. In this article, three psychiatrists give their different viewpoints on the current status of treatment efforts for schizophrenia in the developing world and the measures that can be taken to increase the proportion of patients receiving treatment.

## Abstract 2

Three psychiatrists give their different viewpoints on the current status of treatment efforts for schizophrenia in the developing world.

## Vikram Patel's Viewpoint: Non-Specialist Community Health Workers Should Play a Key Role in Delivering Care

Although schizophrenia is relatively rare, it is also arguably the most severe mental disorder. In many individuals, the disorder runs a chronic and relapsing course, leading to progressively worsening disability, loss of livelihoods and social networks, and increased risk of discrimination and human rights abuse. To consider what might be the best approach for treating schizophrenia in low- and middle-income countries (LAMIC), we must first address three questions: What is the burden of this disorder? What are the resources available for care? And what is the evidence base for the treatment of this disorder in LAMIC?

Assuming that the point prevalence of schizophrenia in LAMIC is the median figure reported in a recent systematic review—4.6 per 1,000 population [1]—and that the population of LAMIC is 5.3 billion [2], then about 25 million people with schizophrenia live in LAMIC. The health systems of LAMIC are woefully unprepared to address the myriad health and social needs of people with schizophrenia; in most parts of LAMIC, there is less than one qualified mental health professional for half a million to a million people [3] (which will include about 2,500 to 5,000 people with schizophrenia). Therefore most people with schizophrenia in LAMIC probably receive little or no formal care.

What impact does this lack of care have on patients' lives? A recent household study from Mozambique reported that up to half of the patients with psychotic disorders were reported by key informants in their households to be currently in poor health [4]. Traditional medicine was by far the most common type of health care accessed. Lack of services contributes to delayed treatment, which in turn leads to poorer long-term outcomes [5], higher direct and indirect costs of treatment with antipsychotic drugs [6], and increasing mortality [7–9].

Thus, the lack of evidence-based care, exacerbated by rapid changes in social and economic conditions in less developed countries that compromise the ability of informal systems to care for people with schizophrenia [9], represents a looming mental health crisis in these countries. Despite these scarce resources, there is now growing evidence that antipsychotic drugs and community-based, family-focused interventions are effective treatments in LAMIC [10]. The latter help reduce stigma, improve adherence to medication, and strengthen social integration.

How can these treatments be delivered in low-resource settings? The most appropriate model of care is a community-based program that is affordable, feasible, acceptable, and evidence based. Who are the key health professionals needed to deliver such a model? Given the scarce specialist resources in LAMIC, the lion's share of the service delivery would need to be the responsibility of non-specialist health workers. Indeed, the front line of the community mental health-care system need not even be represented by health workers at all, but may be made up of people who live in the community and are trained to provide a range of family- and community-based interventions.

Such care models are now being implemented by a number of community-based organizations such as Basic Needs (a mental health non-governmental organization operating in a number of developing countries and based in the United Kingdom), and have been shown in quasi-experimental designs to be superior to simple outpatient care on clinical and functional outcomes [11]. These programs still require a health practitioner—who could be an appropriately trained physician or nurse practitioner—with sufficient skills in diagnosis and drug treatment. The role of specialists may need to be carefully redefined; where available, outreach services provided by specialists in collaboration with local nongovernmental organizations are a clinically effective and cost-effective approach that produces significant improvements in functioning and decreased burden on the family [12]. In many places, however, the most feasible role for specialists might be to design programs, build capacity of non-specialist health workers, and supervise the quality of care delivered.

The first step in a population-based model must be to improve awareness about the disorder and the service. The community mental health worker needs to establish close networks with members of grass-root, health service, and social welfare organizations. Next, the community mental health worker must identify probable cases of schizophrenia, preferably as early as possible after the onset of the disorder. The ideal model for case identification is likely to be through the use of key informants (individuals who are familiar with the health status of members of the community they live in), a cost-effective method for identifying mental disorders in LAMIC [13]. Then the skilled health practitioner should make the diagnosis and initiate drug treatment. Health practitioners must also play close attention to the physical health needs of their patients, given that physical health problems are common in people with schizophrenia and are often neglected, contributing to higher mortality [14]. Health practitioners should also have access to an inpatient unit for the short-term care of patients who are acutely disturbed.

After this predominantly clinical phase, the focus of care shifts back to the community, involving three strategies: (1) Strengthening opportunities for paid employment; (2) building individual and family capacity to cope with the disorder (e.g., to support medication adherence); and (3) ensuring that the costs of long-term care are at least partly borne by an equitable financing system, such as a voucher system, insurance plan, or fixed monthly payments. The role of the community worker may extend to care for people with other severe neuropsychiatric disorders, such as mental retardation and epilepsy, which share many characteristics with schizophrenia (e.g., chronicity, disability, stigma, and loss of livelihood), and are also more likely to occur together. Community-based residential care will still be needed for the few individuals who are severely disabled and cannot continue to live either independently or with their families.

The model that I have outlined is not a pipe dream. It is an affordable prescription for a commitment to ensure that people with schizophrenia receive the basic minimum package of evidence-based care in LAMIC, care that meets their human rights.

## Saeed Farooq's Viewpoint: Directly Observed Therapy (DOTS) Is an Approach Worth Testing

In developing countries, treatment for schizophrenia is limited mostly to acute episodes and seldom involves primary care physicians. Developing countries typically spend less than 1% of their health budget on mental health [15], and one of the consequences of this under-spending is a high prevalence of untreated schizophrenia in the form of undetected as well as inadequately and partially treated cases. In Bihar, one poor state in India, there are more people suffering from schizophrenia than in the whole of North America [16]. The high prevalence in developing countries is partly explained by the predominantly younger population of the developing world (schizophrenia is a disease of young adults).

One approach to tackling the burden of untreated schizophrenia in low-income countries that may prove effective is directly observed therapy. This approach is the cornerstone of current global efforts to tackle tuberculosis (TB). Successful TB treatment, and the prevention of multi-drug resistant TB, requires long-term therapy and high adherence rates. The internationally recommended TB strategy known as DOTS (Directly Observed Therapy, Short-Course) [17] has two essential components: (1) a regular uninterrupted supply of a standardized treatment regimen of six to eight months chemotherapy, and (2) its administration under the supervision of a health worker or trained close relative who watches and records the patient swallowing the correct dose of drugs. A discussion of all five components of DOTS and its implementation is found in [17].

The DOTS strategy has led to remarkable improvements in TB control in many developing countries [18,19]. The World Bank considers DOTS to be one of the most cost-effective health interventions, more cost-effective than self-administered treatment [20,21]. I would argue that the principles underlying the DOTS strategy could form the basis for an effective public health intervention to cope with the burden of schizophrenia in developing countries.

Providing a regular supply of antipsychotic medication and supervising its administration may be one mechanism for addressing the enormous public health burden of long-term untreated psychosis in developing countries. This burden includes increased co-morbid substance abuse, suicide, treatment resistance, impaired cognitive and neuropsychological function, offending behavior, vocational failure, and overall poor outcome [22]. In addition, the strongest predictor of relapse is discontinuation of medication, which increases the relapse risk 5-fold [23]. Even a very short break from taking medication (just one to ten days over a one-year period) is significantly associated with increased risk of hospitalization (odds ratio 1.98, 95%, confidence interval 1.27–3.25) [24].

Long-term antipsychotic medication for treating schizophrenia in developing countries is a cost-effective intervention. Out of 20 recommendations for optimal treatment suggested by the Schizophrenia Patient Outcome Research Team, a research team funded by the US National Institute of Mental Health, 14 were related to pharmacological interventions [25]. These interventions were also rated highest on ease of implementation and can be implemented in developing countries, provided access to the drugs is ensured through a programme akin to DOTS. The cost of antipsychotic medication, including recent atypical drugs, is surprisingly very low in many developing countries [16].

We surely owe it to the families of patients with schizophrenia in poor countries to provide free drugs. The families have largely subsidized schizophrenia treatment for society and the state at large by providing the social, psychological, residential, and occupational support that constitutes the major proportion of the cost of treatment for this disorder. Provision of free drugs to these patients as a part of “DOTS-type” programme would help to share this burden in a small but very significant way.

Drug treatment for schizophrenia is likely to be more effective if its administration is supervised. Such a system of supervision is feasible in low-income settings in view of the family's integral involvement in the patient's care. About 60% of patients with schizophrenia may fail to adhere to their treatment [26], in part because the disease itself leads to impaired insight and cognitive functioning. Approaches that are broadly similar to DOTS, entrusting the monitoring of drug compliance to a relative, have been found to be effective in improving treatment adherence for schizophrenia in developing countries [27,28,11].

I suggest that patients with schizophrenia in the developing world be supplied free access to drugs for two years under close supervision. This will help to overcome non-adherence during the period of the illness (i.e., the first two years) that has been shown to be the strongest predictor of long-term outcome and disability [29]. Indeed, a recent systematic review of interventions to address non-adherence in people with schizophrenia also recommended that clinical interventions targeting non-adherence should continue for at least 18 months [30]. In our pilot project, we found that a supervised treatment approach was associated with greater adherence rates. Encouraged by this finding, we have started a randomized controlled trial of “Supervised Treatment of Outpatient Schizophrenia (STOPS)” to evaluate its effectiveness versus usual care (further details available from author on request and at http://www.clinicaltrials.gov/ct/show/NCT00392249?order=1).

Would direct observation of schizophrenia treatment be overly coercive? In view of the wide treatment gap, there will always be a large population of those with schizophrenia who will be willing to take treatment under supervision, and they must be provided with effective interventions. Moreover, in view of the present poor state of mental health legislation in many developing countries, patients with untreated schizophrenia are likely to suffer from much greater human rights abuses than those who are treated.

Effective treatments for schizophrenia have neither been applied optimally nor advocated as public health interventions in developing countries. A public health intervention for schizophrenia modeled on DOTS may lead to greater awareness of the benefits of treatment and may encourage untreated patients to seek help earlier in the course of their illness. Promoting awareness of the benefits of treatments may help to reduce the stigma of schizophrenia, just as the advent and effective implementation of anti-tuberculosis treatment did for TB. A community intervention for schizophrenia based on the principles of DOTS could also help to put mental health strongly on the public health agenda.

### Acknowledgments

SF is grateful to Prof. Arshad Javed and Dr. Christopher Potter for helpful comments and suggestions on earlier version of the article, and to Dr. Zahid Nazar and Dr. Javed Akhter for their participation in the pilot project and the randomized controlled trial.

## R. Thara's Viewpoint: We Must Tackle Stigma by Offering Proven Treatments

The incidence and prevalence of schizophrenia does not vary widely enough across the world to merit markedly different local treatment approaches or programmes [1,31]. However, the reality is that there are widespread differences in the treatment that people with schizophrenia receive in different parts of the world. Understanding the reasons for these differences is the key to improving the care of people with schizophrenia in developing countries, including India, where I work.

An important reason underscoring these differences is that many developing countries have far too few mental health professionals [3], reflecting the very low priority accorded to mental health by many governments. In many low-income countries, there is an urgent need to improve and expand mental health services.

Community care in India is almost synonymous with family care. There are no organized community-based programmes for people with chronic mental illness. The commonest site of treatment is the mental hospital, many of which are large and isolated, with little contact with the community they serve. Efforts are under way to improve the conditions of many of these hospitals. While the number of general hospital psychiatry beds has increased in the last decade, the total number is still grossly inadequate. Most private psychiatrists are located in urban areas. There are very few non-governmental organizations, and these are largely concentrated in the southern part of the country.

India's National Mental Health Programme did envisage the diffusion of mental health skills to primary health-care centres at the village and district levels, and the integration of mental health with primary care. However, poor monitoring and lack of coordination with the local state governments meant that such diffusion and integration efforts were not implemented, with the exception of a few sporadic programmes.

In India, people with chronic mental illness do not generally receive any welfare benefits, except for some minor benefits in just a few areas. Medical insurance seldom covers treatment of mental disorders. The result is that families have to bear the entire costs of the treatment and ongoing care of these patients. A heavy financial, physical, and emotional burden is therefore imposed upon family members. Indeed, over 90% of patients with schizophrenia live at home with their families [32]. Such family involvement is not merely a result of close kinship ties, but is also due to inadequate treatment facilities. Religious and traditional modes of intervention are still widely practiced, especially in rural areas, where mental health services are almost non-existent. Families are equal partners in all stages of intervention, be it choice of a drug, detection of side effects, or early symptoms of relapse, ensuring compliance with medication and supporting the affected family member through life events such as jobs or marriages.

Stigma, and the presence of competing and conflicting explanatory models of mental illness (often based on “magico-religious” beliefs), have also contributed to the non-use of existing treatment facilities. A study that we conducted at the Schizophrenia Research Foundation in Chennai found that women with schizophrenia were more stigmatized than men with schizophrenia, and that female caregivers were more sensitive to stigma than male caregivers. Being single or divorced compounded the problem of stigma even further [33]. Stigma is an all-encompassing phenomenon and a profound barrier to effective help seeking.

In terms of the availability of medication for treating schizophrenia in India, both first and second generation antipsychotics (risperidone, olanzapine, clozapine, quetiepine, and sulpiride) are available at fairly low prices. Both groups of drugs are being used as first-line medication. In my clinical experience I have found that Indian patients require a much lower dosage of medicines than patients in the West, in both the acute and maintenance phases of treatment. However, more research is needed to document whether there are indeed different dosage requirements in Indian patients and, if there are, to critically address the actual reasons for this difference. Unfortunately, a large proportion of patients in both rural and urban settings remain untreated [34,35]. Even when patients are prescribed medication, non-compliance during the symptomatic phase is common in India. In our study of patients attending an urban outpatient care center, we noted that when the patients were acutely ill and refused to take medication, in half the cases the families administered medication to them without the patients' knowledge, under the supervision of the psychiatrist [32].

Given all of these different factors affecting the current management of schizophrenia, what can be done to improve such care? Ensuring that patients receive effective treatments promises to be the best antidote to stigma. When patients' conditions improve, especially in the restoration of their social functioning, the community's explanatory model of schizophrenia often shifts from a magico-religious to a medico-social viewpoint. The National Mental Health Programme will have to be scaled up to ensure that mental health care reaches the masses. At the same time, the government should plan and implement awareness programmes for schizophrenia all over the country, as it did for leprosy and tuberculosis.

Efforts will simultaneously have to be made to prevent Indian psychiatrists from going abroad, since their numbers have dwindled rapidly in the last few years. More psychiatry tuition in the undergraduate medical curriculum is also critical. Unless all these measures are implemented urgently and in a comprehensive manner, mental health care will continue to languish in the backyards of the health-care system.

## Abbreviations

DOTS: directly observed therapy, short-course
LAMIC: low- and middle-income countries
TB: tuberculosis

## References

[pmed-0040159-b001] Saha S, Chant D, Welham J, McGrath J (2005). A systematic review of the prevalence of schizophrenia. PLoS Med.

[pmed-0040159-b002] Population Reference Bureau (2006). 2006 world population data sheet. http://www.prb.org/Content/NavigationMenu/PRB/PRB_Library/Data_Sheets/Data_Sheets.htm.

[pmed-0040159-b003] World Health Organization (2005). Mental health atlas. http://globalatlas.who.int/globalatlas/default.asp.

[pmed-0040159-b004] Patel V, Simbine A, Soares I, Weiss H, Wheeler E (2007). Prevalence of severe mental and neurological disorders in Mozambique: A household key informant survey in rural and urban settings. Lancet.

[pmed-0040159-b005] Oosthuizen P, Emsley RA, Keyter N, Niehaus DJ, Koen L (2005). Duration of untreated psychosis and outcome in first-episode psychosis. Perspective from a developing country. Acta Psychiatr Scand.

[pmed-0040159-b006] Grover S, Avasthi A, Chakrabarti S, Bhansali A, Kulhara P (2005). Cost of care of schizophrenia: A study of Indian out-patient attenders. Acta Psychiatr Scand.

[pmed-0040159-b007] Kebede D, Alem A, Shibre T, Negash A, Deyassa N (2005). Short-term symptomatic and functional outcomes of schizophrenia in Butajira, Ethiopia. Schizophr Res.

[pmed-0040159-b008] Kurihara T, Kato M, Reverger R, Tirta IG (2005). Eleven-year clinical outcome of schizophrenia in Bali. Acta Psychiatr Scand.

[pmed-0040159-b009] Patel V, Cohen A, Thara R, Gureje O (2006). Is the outcome of schizophrenia really better in developing countries?. Rev Bras Psiquiatr.

[pmed-0040159-b010] Hyman S, Chisholm D, Kessler R, Patel V, Whiteford H, Jamison DT, Breman JG, Measham AR, Alleyne G, Claeson M (2006). Mental disorders. http://www.dcp2.org/pubs/DCP.

[pmed-0040159-b011] Chatterjee S, Patel V, Chatterjee A, Weiss H (2003). Evaluation of a community based rehabilitation model for chronic schizophrenia in a rural region of India. Br J Psychiatry.

[pmed-0040159-b012] Srinivasa Murthy R, Kishore Kumar KV, Chisholm D, Thomas T, Sekar K (2005). Community outreach for untreated schizophrenia in rural India: A follow-up study of symptoms, disability, family burden and costs. Psychol Med.

[pmed-0040159-b013] Isaac MK, Kapur RL (1980). A cost-effectiveness analysis of three different methods of psychiatric case finding in the general population. Br J Psychiatry.

[pmed-0040159-b014] Mitchell AJ, Malone D (2006). Physical health and schizophrenia. Curr Opin Psychiatry.

[pmed-0040159-b015] World Health Organization (2003). Investing in mental health. http://www.who.int/mental_health/media/investing_mnh.pdf.

[pmed-0040159-b016] Adams C, Prathap T, Ciutinho ESF, Stroup TS (2006). The schizophrenia drug-treatment paradox: Pharmacological treatment based on best possible evidence may be hardest to practice in high-income countries. Br J Psychiatry.

[pmed-0040159-b017] World Health Organization (1999). What is DOTS? A guide to understanding the WHO-recommended TB control strategy known as DOTS. http://whqlibdoc.who.int/hq/1999/WHO_CDS_CPC_TB_99.270.pdf.

[pmed-0040159-b018] Squire SB, Tang S (2004). How much of China's success in tuberculosis control is really due to DOTS?. Lancet.

[pmed-0040159-b019] China Tuberculosis Control Collaboration (2004). The effects of tuberculosis control in China. Lancet.

[pmed-0040159-b020] Moore RD, Chaulk CP, Griffiths R, Cavalcante S, Chaisson RE (1996). Cost effectiveness of directly observed versus self-administered therapy for tuberculosis. Am J Resp Crit Care Med.

[pmed-0040159-b021] Floyed K, Wilkinson D, Gilk C (1997). Comparison of cost effectiveness of directly observed therapy (DOT) and conventionally delivered treatment for tuberculosis: Experience from rural South Africa. BMJ.

[pmed-0040159-b022] Lieberman JA, Fenton WS (2000). Delayed detection of psychosis: Causes, consequences, and effect on public health. Am J Psychiatry.

[pmed-0040159-b023] Robinson D, Woerner MG, Alvir JMJ, Bilder R, Goldman R (1999). Predictors of relapse following response from a first episode of schizophrenia or schizoaffective disorder. Arch Gen Psychiatry.

[pmed-0040159-b024] Weiden PJ, Kozma C, Grogg A, Locklear J (2004). Partial compliance and risk of rehospitalization among California Medicaid patients with schizophrenia. Psychiatr Serv.

[pmed-0040159-b025] Lehman AF, Kreyenbuhl J, Buchanan RW, Dickerson FB, Dixon LB (2004). The Schizophrenia Patient Outcomes Research Team (PORT): Updated treatment recommendations 2003. Schizophr Bull.

[pmed-0040159-b026] Kampman O, Lehtinen K (1999). Compliance in psychoses. Acta Psychiatrica Scandinavica.

[pmed-0040159-b027] Qui F, Lu S (1994). Guardianship networks for rural psychiatric patients. Br J Psychiatry.

[pmed-0040159-b028] Murthy RS, Kishore KVK, Chisolm D, Thomas T, Seskar K (2005). Community outreach for untreated schizophrenia in rural India: A follow-up study of symptoms, disability, family burden and costs. Psychol Med.

[pmed-0040159-b029] Harrison G, Hooper K, Craig T, Laska E, Siegel C (2001). Recovery from psychotic illness: A 15- and 25- years international follow up study. Br J Psychiatry.

[pmed-0040159-b030] Zygmunt A, Olfson M, Boyer CA, Mechanic D (2002). The interventions to improve medication adherence in schizophrenia. Am J Psychiat.

[pmed-0040159-b031] McGrath J, Saha S, Welham J, El Saadi O, MacCauley C (2004). A systematic review of the incidence of schizophrenia: The distribution of rates and the influence of sex, urbanicity, migrant status and methodology. BMC Med.

[pmed-0040159-b032] Srinivasan TN, Thara R (2002). Management of medication non-compliance in schizophrenia by the families in India. Schizophr Bull.

[pmed-0040159-b033] Thara R, Srinivasan TN (2000). How stigmatising is schizophrenia in India?. International J Social Psychiatry.

[pmed-0040159-b034] McCreadie RG, Thara R, Kamath S, Padmavathy R, Latha S (1996). Abnormal movements in never-medicated Indian patients with schizophrenia. Br J Psychiatry.

[pmed-0040159-b035] Padmavati R, Rajkumar S, Srinivasan TN (1998). Schizophrenic patients who were never treated—A study in an urban Indian community. Psychol Med.

